# Guided immobilisation of single gold nanoparticles by chemical electron beam lithography

**DOI:** 10.3762/bjnano.4.39

**Published:** 2013-05-31

**Authors:** Patrick A Schaal, Ulrich Simon

**Affiliations:** 1 Institute of Inorganic Chemistry and JARA – Fundamentals of Future Information Technology, RWTH Aachen University, Landoltweg 1, D-52056 Aachen, Germany

**Keywords:** 2D pattern, indium tin oxide (ITO), positioning, SAM, self-assembly

## Abstract

The fabrication of periodic arrays of single metal nanoparticles is of great current interest. In this paper we present a straight-forward three-step procedure based on chemical electron beam lithography, which is capable of producing such arrays with gold nanoparticles (AuNPs). Preformed 6 nm AuNPs are immobilised on thiol patterns with a pitch of 100 nm by guided self-assembly. Afterwards, these arrays are characterised by using atomic force microscopy.

## Introduction

Periodic arrays of nanometre-sized metal structures hold great promise for future applications, e.g., in nanoelectronics [1–4] or in biohybrid devices [5–6]. The most common technique to generate such structures is the evaporation of a thin metal film through a resist mask structured by electron beam lithography (EBL) or other lithographic techniques [1,7]. In general, these fabrication techniques involve five or more processing steps, including formation, patterning and development of resist films, metal evaporation/sputtering and lift-off, whereby feature sizes rarely go beyond the 10 nm threshold [1]. Depending on the chosen substrate, e.g., SiO_2_, additional metal layers such as Ti are needed as adhesive layers.

In order to overcome this threshold and to facilitate the processing, alternative approaches have been developed, which utilise the self-assembly capabilities of chemically tailored metal nanoparticles. Amongst others, Enderle et al. demonstrated very recently the formation of gold nanodots by self-assembly of micelles loaded with HAuCl_4_ and subsequent reduction by hydrogen plasma [8]. Such assembly protocols are more facile, but are limited to the formation of self-forming periodic patterns, which are typically of hexagonal symmetry [8–10].

In order to increase the structural variability, guided immobilisation of single AuNPs by chemically structured surfaces has been introduced [11–12]. Appropriate surfaces can be obtained either by resist-based EBL and subsequent etching [11] or by soft lithographic techniques such as nanoimprint lithography [12]. As an example, Onses et al. demonstrated the fabrication of very precise patterns of single 13 nm AuNPs with pitches around 80 nm very recently [11]. However, both techniques are technically demanding and require several processing steps or need prefabricated molds and are, therefore, not easily adaptable to new designs.

Very recently, we reported the formation of electrically conducting nanopatterns formed by chemical EBL (CEBL) [13]. Therefore, we formed a chemically patterned surface by local reduction of the terminal SO_2_X groups of self-assembled monolayers (SAMs) by means of an electron beam [14]. These structured SAMs guided AuNP immobilisation through covalent binding. A subsequent metallisation step enabled the formation of conducting nanopatterns in the 100 nm regime. Compared to resist-based EBL with five or more processing steps, the pattern formation was achieved in just three steps (SAM preparation, irradiation, and immobilisation), however, with significantly lower fidelity. Therefore, it would be highly desirable to develop this method further to take full advantage of the structural variability that arises from EBL and the high degree of control over size and shape of chemically tailored AuNPs to deposit ideally individual AuNPs in any type of periodic or aperiodic pattern.

In order to make new steps in this direction, in this work, we present the local reduction of sulfonic acid terminated SAMs into thiol-terminated SAMs by CEBL on electron-transparent SiO_2_ membranes, which enabled us to analyse the site-selective immobilisation of AuNPs by scanning electron microscopy in transmission (SEM-T) and by atomic force microscopy (AFM). Based on these analyses, we were able to optimise the process yielding periodic patterns of single 6 nm AuNPs.

## Results and Discussion

### Generation of thiol groups on thin Si/SiO_2_ membranes

**Figure 1:**
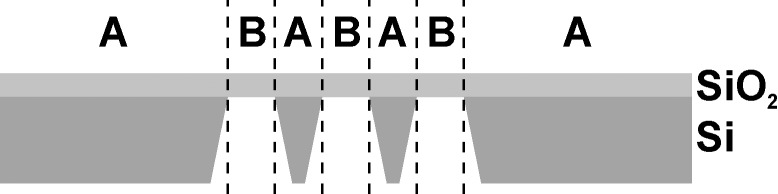
Schematic drawing of used membranes with 100 μm thick Si grid and a 40 nm layer of electron-transparent SiO_2_.

Following the protocol we published previously [13], we studied the reduction of 2-(4-chlorosulfonylphenyl)ethyltrichlorosilane (CSPETCS) SAMs on top of electron-transparent SiO_2_ layers. For this we used SiO_2_ membranes, which are commonly used in SEM-T and TEM experiments (Figure 1). Within region **A** of these substrates the vertical layer composition is 100 μm of Si covered with 40 nm of SiO_2_. In contrast to this, in region **B** the 40 nm SiO_2_ layer is suspended without any support. CSPETCS SAMs were fabricated by wet-chemical silanisation in dry toluene within both regions **A** and **B**. Thereby, CSPETCS SAMs with a thickness down to 1.3 ± 0.1 nm could be fabricated. Upon irradiation with electrons these monolayers can be locally reduced converting the top sulfonic acid group into a thiol group [13].

**Figure 2:**
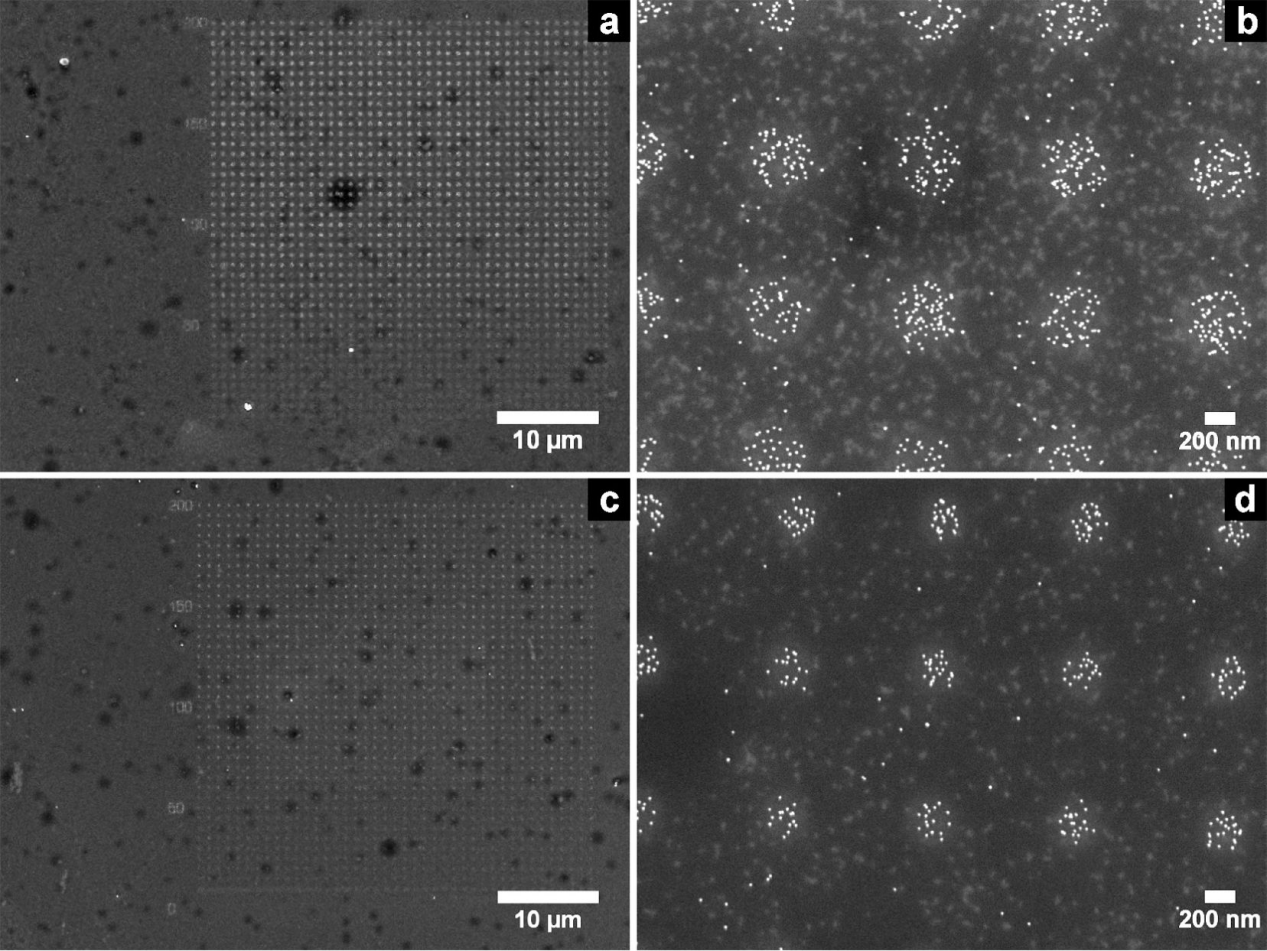
SEM pictures of irradiated CSPETCS layer on a Si/SiO_2_ substrate (100 μm/40 nm) after incubation with a solution of 16 nm AuNPs. Two patterns with circular structures of different intended diameter were used: 400 nm (a,b) and 200 nm (c,d).

First, we irradiated the CSPETCS layer in region **A** with electrons at acceleration voltages (EHT) of 2 kV and a base dose of 50 μC·cm^−2^ (see Supporting Information File 1 for a theoretical calculation, primary electron pathways, and estimated influence upon irradiation dose) using CAD-designs with circles of 400 nm and 200 nm, respectively (cf. Figure S1 and Figure S2 within Supporting Information File 1 for details). The exposed substrates were then incubated with a solution of 16 nm AuNP at pH 4.7. Figure 2 shows exemplary SEM pictures of the incubated patterns with intended structure diameters of 400 nm (a,b) and 200 nm (c,d). The actual average diameters were determined to 420 ± 20 nm and 230 ± 10 nm by statistical analysis. In accordance with previous experiments, this process is capable of fabricating AuNP patterns with a selective deposition in irradiated areas on silanised SiO_2_ surfaces of 40 nm thickness.

**Figure 3:**
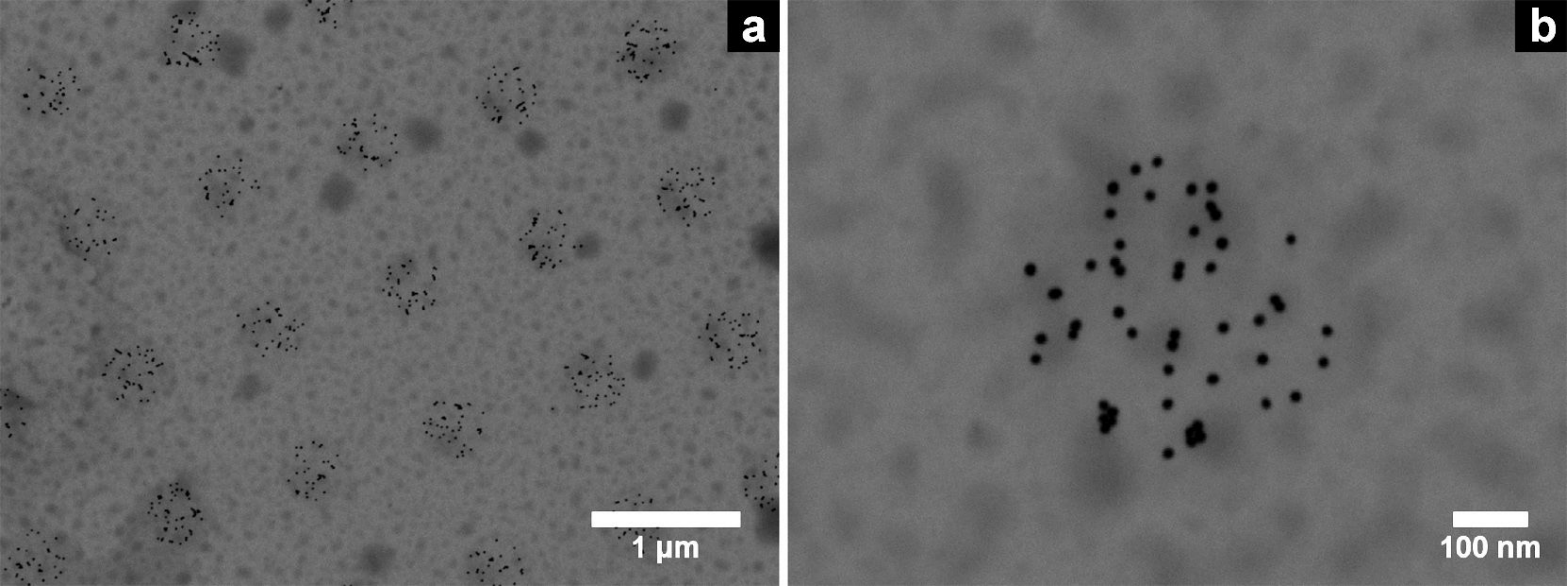
SEM-T micrographs of CSPETCS silanised SiO_2_ TEM windows after irradiation and incubation with a solution of citrate-stabilised 16 nm AuNPs. The intended and the actual spot diameters are 400 nm and 400 ± 20 nm, respectively.

In order to examine the possibility of structuring the freely suspended SiO_2_ windows, we performed the same experiments within region **B** (c.f. Figure 1). After incubation with a solution of 16 nm AuNPs the generated patterns were imaged by SEM-T. Two exemplary images are shown in Figure 3. The immobilised AuNPs are well silhouetted against the electron-transparent SiO_2_ background. The actual spot diameter of 400 ± 20 nm matches the intended one of 400 nm. The average particle density within the irradiated spots is ρ_NP_(SH) = 300 ± 30 NP/μm^2^ and significantly higher than outside the irradiated areas with ρ_NP_(SO_2_X) < 10 NP/μm^2^.

### Incubation of thiol patterns with 6 nm AuNP

**Figure 4:**
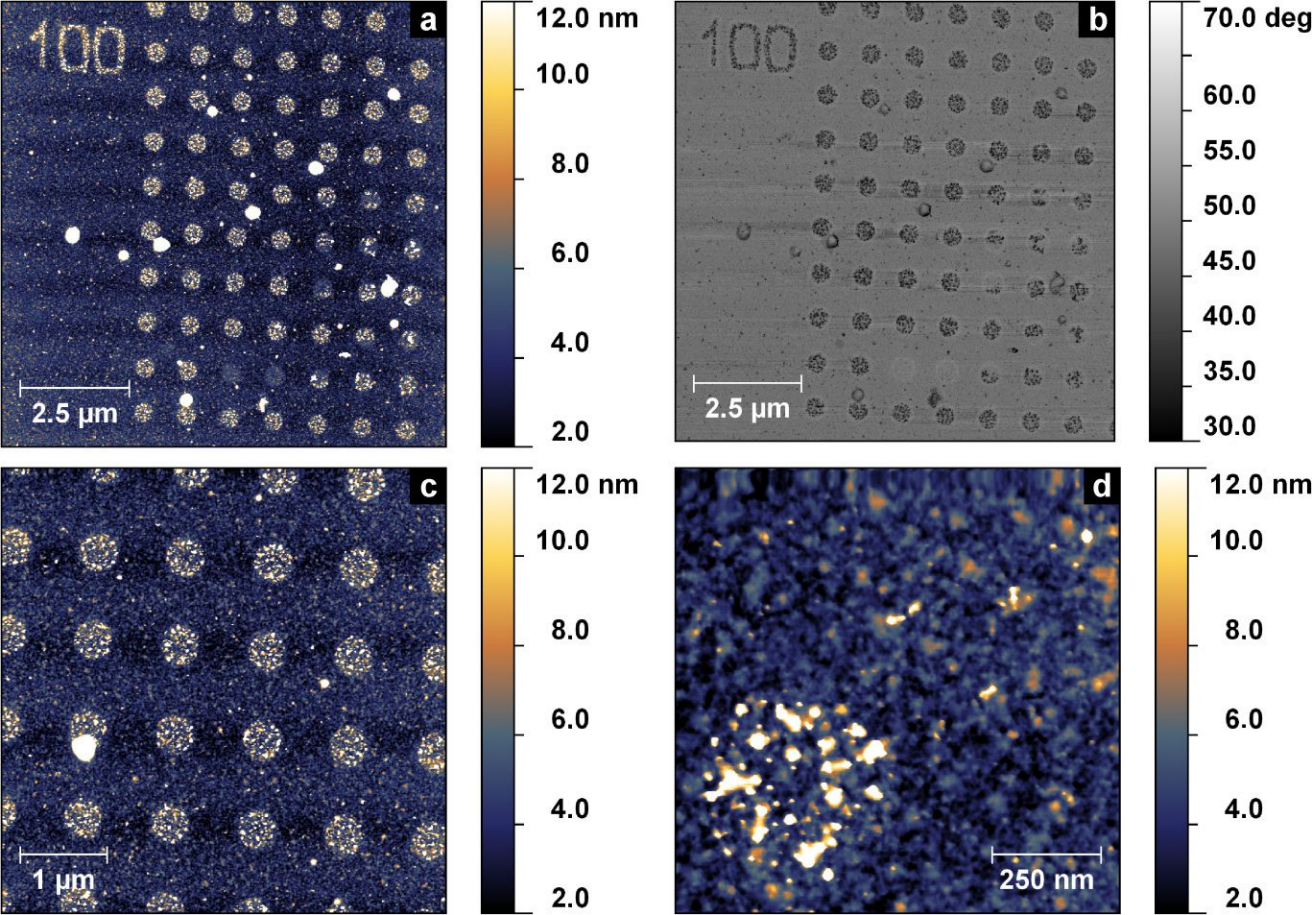
Height (a,c,d) and phase (b) measurements via AFM of an irradiated CSPETCS monolayer incubated with 6 nm AuNP.

Attempts to generate smaller structures, e.g., circles with diameter *d* < 200 nm, resulted in randomly and incompletely covered structures due to the decreasing structure-to-particle size ratio (*d*_S_/*d*_NP_) and the large distance between immobilised AuNPs (see Figure S4 and Figure S5 within Supporting Information File 1 for an example). In order to increase the coverage of these thiol patterns as well, we used 6 nm AuNPs with a larger *d*_S_/*d*_NP_. Figure 4 shows measurements by atomic force microscopy (AFM) of the structured surface (EHT = 2 kV and base dose of 10 μC·cm^−2^) after incubation. The density of immobilised particles within the irradiated structures is greater than 480 ± 30 NP/μm^2^ and, therefore, slightly higher than the particle density of the previously shown structures.

**Figure 5:**
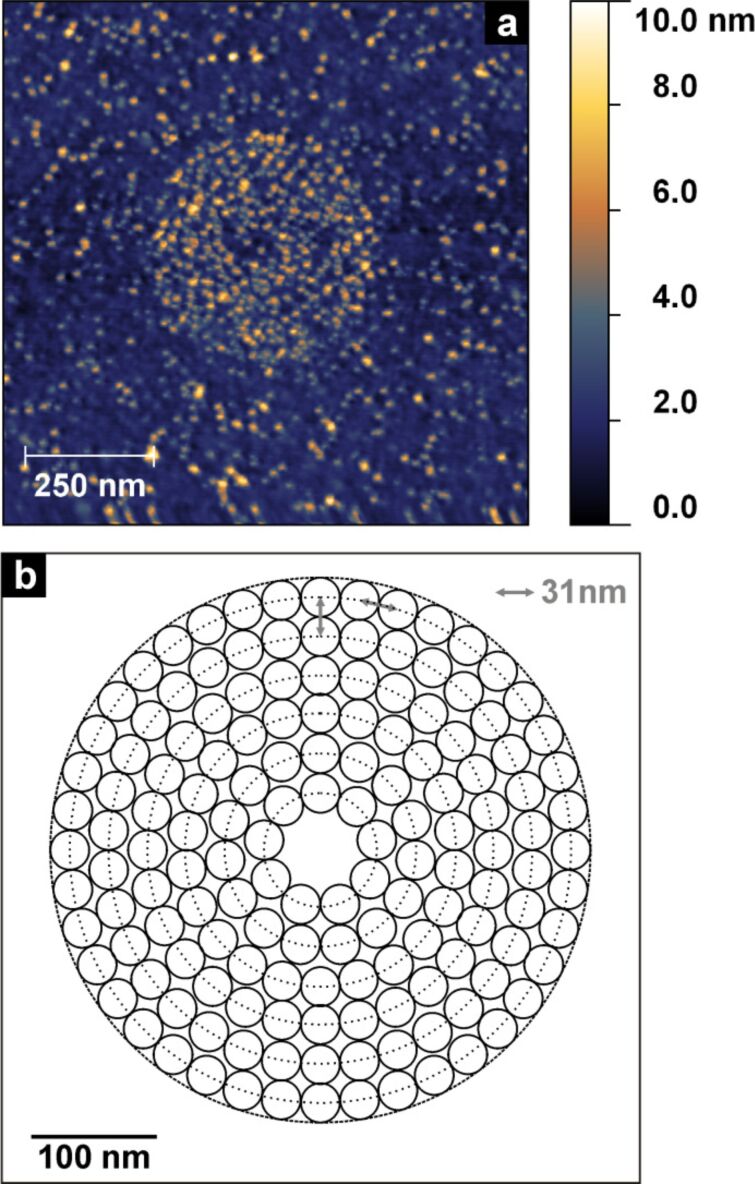
Super structure of immobilised 6 nm AuNPs on a circular structure written by CEBL (a) and possible grating pattern of a circle to be written with a beam step size of 31 nm (b).

Upon closer inspection, we found that the written and incubated structures exhibited regular super structures (Figure 5a), that resemble the used grating pattern (Figure 5b). The grating is performed on the basis of a pre-defined spacing *S* (also called beam step size), in this case 31 nm. The circular structure to be written is subdivided into concentric rings with decreasing diameter by a value of 2*S* (i.e., spacing of *S*). On the perimeters of these rings individual irradiation spots are placed, which are separated by *S* from each other. In order to expose this grated structure the electron beam dwells on these single spots.

**Figure 6:**
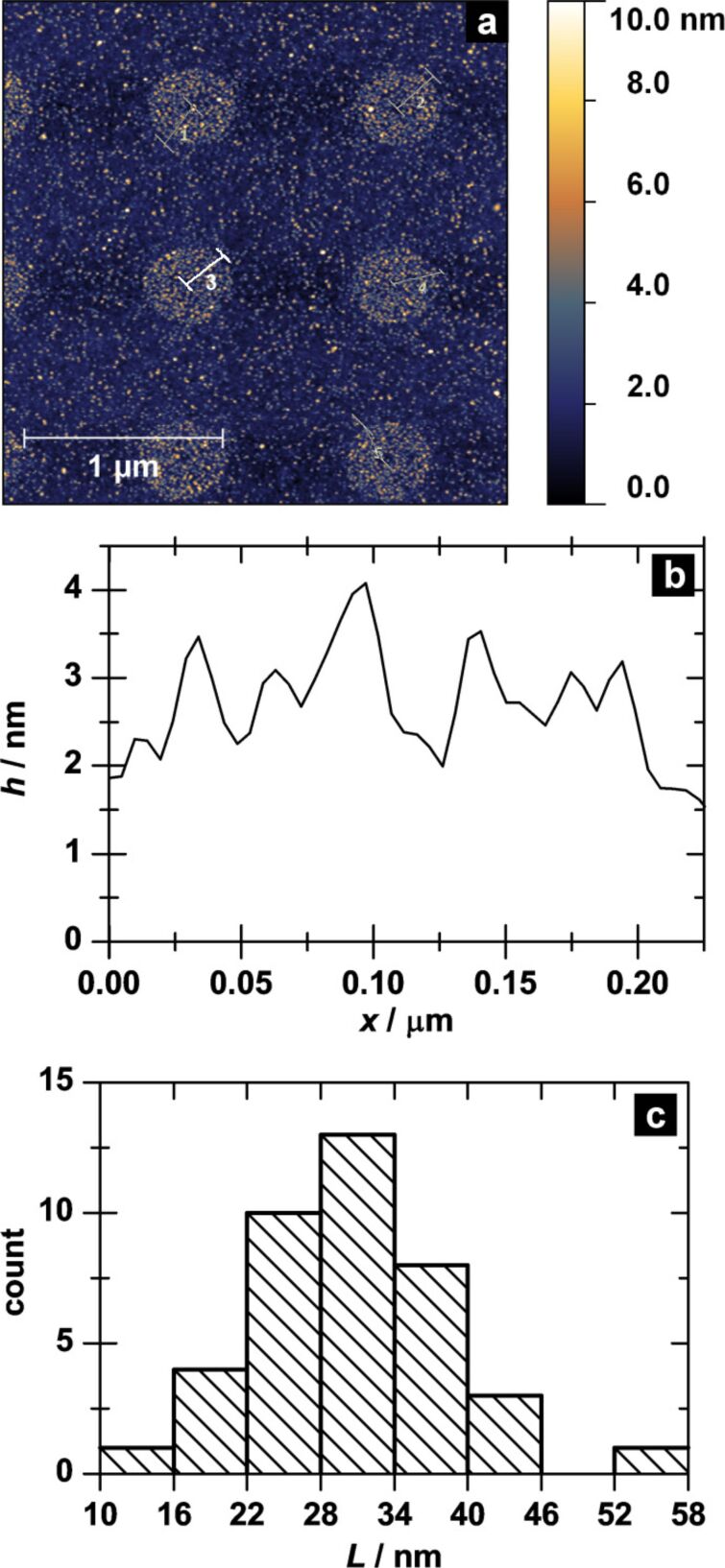
AFM height measurement of a superstructured AuNP pattern (a), exemplary height profile (b) of line 3 in (a), and histogram of the determined peak-to-peak distances in all measured height profiles (c).

To verify a direct correlation between the grating pattern and the generated super structures we measured random height profiles along the cross-sections of the circular structures (Figure 6a). Figure 6b shows an exemplary height profile within the AFM measurement in Figure 6a. From the peak-to-peak distance in this height profile the spacing between the concentric rings can be determined. The average spacing of the individual rings is *L*_r_ = 30 ± 8 nm (cf. histogram of all measured spacings *L*_r_ in Figure 6c) and is, hence, originated by the initial grating spacing *S*. Since the actual diameter of the used Gaussian electron beam is much smaller (approx. 1 nm) than the grating distance, the electron dose applied to the SAM decreases with increasing distance from the grating spots (being lowest in the middle between two spots). The variation of the electron dose within the irradiated structure results in a variation of the thiol density. With decreasing particle size this thiol gradient becomes the driving force for the guided immobilisation into the observed super structure. Theoretical calculations show that the energy of scattered electrons of a 1 nm wide electron beam at 2 kV decreases exponentially with increasing distance from the point of incidence. With respect to a necessary threshold energy of around 10 eV for the first mechanistic step of the CEBL process (DEA, dissociative electron attachment) [15–16], the area that is effectively irradiated (i.e., where thiol groups are generated) is approximately 5–6 nm in diameter. Assuming that the immobilisation of a AuNP is most stable with a maximum contact area between AuNP and SAM, particles exhibit more linking possibilities with increasing size. This results in a significantly lower freedom of displacement for particles that match the spot size (i.e., 6 nm AuNPs in the present study).

Hence, it became possible to assemble single nanoparticles through guided immobilisation on CEBL-structured CSPETCS SAMs. Due to forward scattering of the electron beam within the substrate, primary and secondary electrons are able to exit the substrate outside of the actual area of beam incidence, resulting in SAM exposure outside the irradiated area. This phenomenon results in a higher effective dose of an irradiation point due to all other irradiation points in the near vicinity. A general problem in producing chemical patterns that are fine enough for guided single-particle immobilisation by CEBL is that with decreasing feature size and density, higher electron doses are needed in order to achieve a proper SAM reduction. This effect is also known from conventional lithography techniques and is called the “proximity effect” [17–18]. Unfortunately, higher doses and prolonged exposure times result in blurry and diffuse pattern generation.

**Figure 7:**
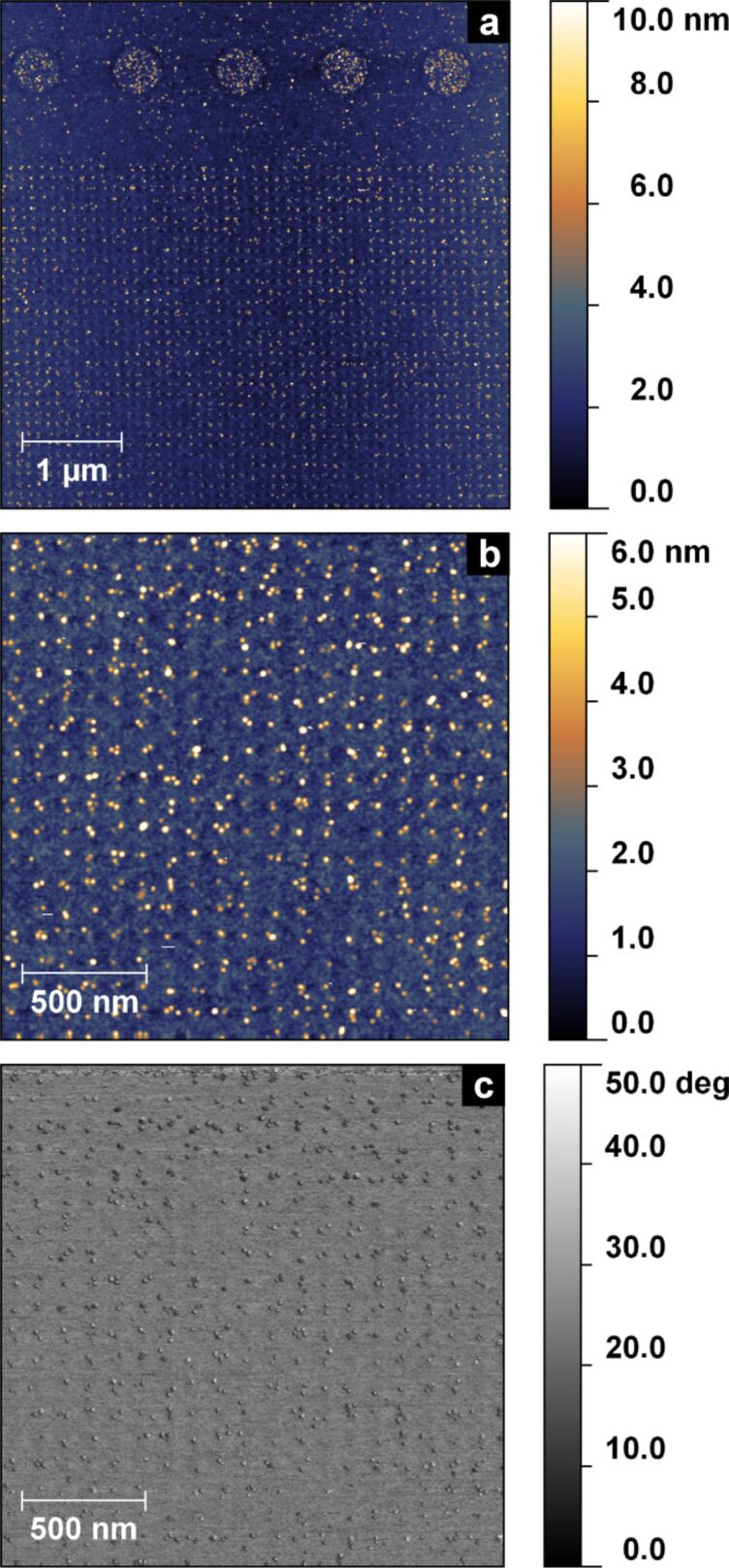
AFM measurements of height (a, b) and phase (c) of a CSPETCS SAM patterned by pointwise irradiation with electrons after incubation with citrate-stabilised 6 nm AuNP.

In order to overcome the need for prolonged exposure we designed a periodic high-density pattern of single irradiation points (cf. Figure S6 in Supporting Information File 1). A CSPETCS SAM was then structured with this pattern using standard CEBL at EHT = 2 kV and a base dose of 10 μC·cm^−2^. After incubation with citrate-stabilised 6 nm AuNP at pH 4.7, we analysed the substrate by AFM. Figure 7a and b show height measurements of the incubated surface.

**Figure 8:**
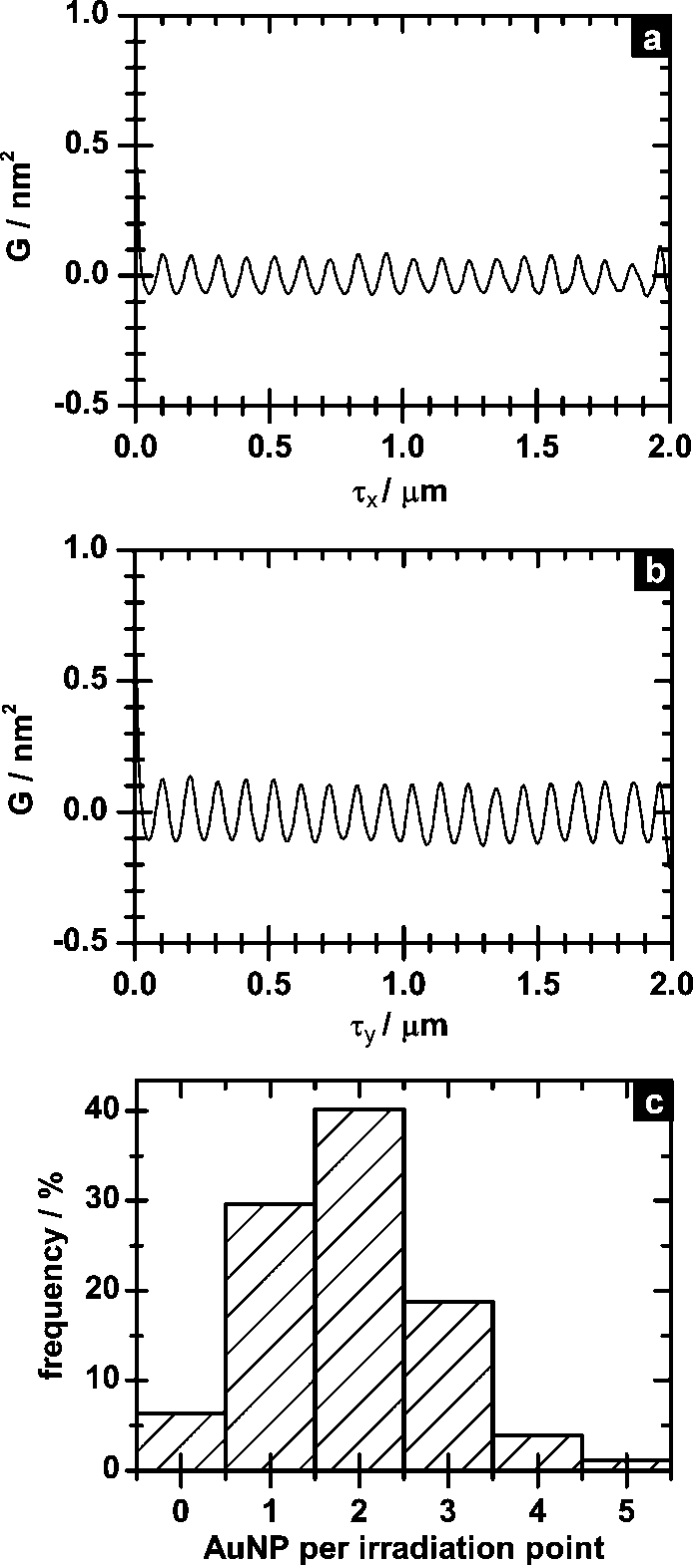
Horizontal (a) and vertical (b) spatial autocorrelation of AFM height measurement in Figure 7b and histogram of the number of AuNPs per irradiation spot (c).

To analyse the precise arrangement of the immobilised AuNP we performed spatial autocorrelation (AC) in the x- and y-direction, the result of which are shown in Figure 8a and b. Both graphs show a high degree of order in both directions with spatial periodicities of Δτ_x_ = 103 ± 3 nm and Δτ_y_ = 103 ± 2 nm. This high periodicity proves the guided immobilisation of AuNP by the fabricated thiol array on the surface. In addition we performed a statistical analysis of the number of AuNPs per irradiation spot. The resulting histogram is shown in Figure 8c. Approximately 70% of all irradiation spots are covered with one or two AuNPs, indicating that the irradiation spots are indeed small enough to provided single-particle immobilisation. Hence, accuracy and immobilisation fidelity are comparable to other approaches presented in the literature with 13 nm AuNPs [11–12]. Since we immobilised significantly smaller AuNPs (6 nm), one can assume that the individual spots generated in our approach are approximately half the size. In addition, our approach benefits from a lower number of processing steps (i.e., three: SAM formation and irradiation followed by AuNP immobilisation).

## Conclusion

In this work we present the fabrication of regular arrays of single AuNPs. These arrays were formed by guided immobilisation of AuNPs through chemically patterned SAMs in three processing steps. Through point-by-point electron irradiation we generated thiol patterns with a periodic pitch of 100 nm in the horizontal and vertical directions. AFM measurements proved that this periodicity was retained after immobilisation of 6 nm AuNPs. Due to the small size of an individual irradiation point approximately 70% of the thiol spots were covered with only one or two AuNPs. Furthermore, we could decorate freestanding 40 nm SiO_2_ layers with AuNPs using the same approach and visualise the obtained AuNP patterns with SEM-T.

In the future, we are interested in transferring the generation of single AuNP arrays to other technically relevant substrates than Si/SiO_2_. Therefore, we conducted initial experiments by fabrication of AuNP patterns on indium tin oxide (ITO) covered foils of polyethylene terephthalate (PET) (see Supporting Information File 1 for the preliminary results), which are quite promising.

## Experimental

### Materials

In this work two different kinds of oxide surfaces were used. Experiments on SiO_2_ surfaces were conducted by using 40 nm thick SiO_2_ membranes on 100 μm Si grids (suitable for TEM analyses) from Plano GmbH. In addition, flexible ITO-coated PET substrates from Aldrich were used. Toluene from Sigma Aldrich was dried over Na/benzophenone ketyl radical and distilled afterwards. 2-(4-Chlorosulfonylphenyl)ethyltrichlorosilane (CSPETCS) was purchased as a 50 wt % solution in toluene from ABCR GmbH. Ethanol (p.a.) was purchased from Grüssing GmbH. Ultrapure water was prepared by using a Purelab Ultra from Elga. Hydrogen tetrachloroaurate(III) trihydrate, sodium borohydride, and trisodium citrate dihydrate were purchased from Sigma Aldrich or Merck. Unless stated otherwise, all substances were used without any further purification.

### Sample cleaning and silanisation

Prior to cleaning ITO-coated PET foils were cut into proper pieces. Both types of substrates (SiO_2_ and ITO) were cleaned in oxygen plasma at *p*(O_2_) = 0.4 mbar, *f* = 40 kHz, and *P* = 100 W, first for 2 min and then for 4 min. In the case of Si/SiO_2_ substrates the initial SiO_2_ thickness was measured by ellipsometry between both plasma cleaning steps. All samples were transferred directly into silanisation solutions after venting and silanised in dry toluene by the Schlenk technique. For this, a stem solution of 10 mM CSPETCS in dry toluene was prepared first. Cleaned samples were put into dried Schlenk tubes filled with 3 mL of dry toluene, and 1 mL of stem solution was added. The tube was then heated to 40 °C for 30 min. Afterwards all samples were thoroughly cleaned with ethanol, dried in a nitrogen stream, and annealed at 130 °C for 15 min.

### E-beam lithography

Lithographic patterns were generated using a scanning electron microscope (Zeiss LEO Supra 35-VP) equipped with an Elphy Plus pattern generator (RAITH, software Elphy Plus version 4). Patterns were exposed at an accelerating voltage of 2 kV and basic electron doses of 10 μC·cm^−2^ and 50 μC·cm^−2^, respectively.

### Synthesis and deposition of AuNP

AuNPs with a diameter of 16 nm were synthesised according to known procedures from Turkevich and Frens by using 0.056 mM of tetrachloroaurate(III) trihydrate and 0.178 mM of trisodium citrate dehydrate [19–21]. Afterwards, the pH value was adjusted to 4.7 by centrifugation and redispersion in 10 mM citrate buffer.

In addition to citrate-stabilised 16 nm AuNPs, we synthesised citrate-stabilised AuNPs with a diameter of 6 nm. These particles were prepared by using a modified procedure reported by Patil et al. [22–24]. First, 50 mL of a stirred 0.5 mM solution of hydrogen tetrachloroaurate(III) was reduced by dropwise addition of 4.5 mL of a 48.5 mM solution of sodium borohydride. The red solution was stirred for 5 min and 2.5 mL of a 50 mM solution of trisodium citrate dihydrate was added as a capping agent. Finally, the solution was stirred for an additional 5 min. In order to adjust the pH value to 4.7, 50 mM citrate buffer was added until the buffer concentration in solution was equal to 10 mM.

Irradiated samples were incubated with this AuNP solution for 45 min up to 60 min in a closed chamber in order to prevent drop evaporation. Finally, the substrates were rinsed with a copious amount of ultrapure water and dried in a nitrogen stream.

### Ellipsometry

To determine the SAM thicknesses ellipsometric measurements were performed by using a NFT I-Elli 2000 imaging ellipsometer equipped with a HeNe laser (λ = 632.8 nm). In order to calculate the film thicknesses we use values of *n* = 3.8650 and *k* = 0.0200 for Si and *n* = 1.4650 and *k* = 0.0000 for silicon dioxide and the organic layer [25–26].

### AFM measurements

AFM measurements were conducted with a Digital Instruments NanoScope IIIa by using super sharp tips SSS-NCH-50 from Nanosensors with small tip diameters of approximately 2 nm and force constants between 10 N·m^−1^ and 130 N·m^−1^. Image processing was performed by using the open-source software gwyddion 2.28 (http://gwyddion.net) [27]. In general, measurements were post-processed with the commands “Level data by mean plane subtraction”, “Correct lines by matching height median” and “Correct horizontal scars (strokes)”. In addition, measurements with an edge length greater than 5 µm or distinct curvature were also corrected with “Remove polynomial background (degree: 2)”.

### Calculation of primary electron paths

Paths of primary electrons in solid substrates were calculated with CASINO v2.48 (monte CArlo SImulation of electroN trajectory in sOlids) [28]. Therefore the following density values were used: ρ(Si) = 2.3290 g·cm^−3^ [29], ρ(SiO_2_) = 2.196 g·cm^−3^ [29], ρ(CSPETCS) = 1.35 g·cm^−3^ (estimation using density values of commercially available solutions).

## Supporting Information

Figures S1, S2 and S6 present CAD-drawings of the patterns used within the CEBL process. Figure S3 discusses theoretical calculations of primary electron pathways within the used substrates. SEM pictures of 100 nm structures incubated with 16 nm AuNPs are shown in Figures S4 and S5. Figure S7 presents preliminary results of AuNP pattern formation on ITO-covered PET foils by this approach.

File 1 Additional CAD-drawings, theoretical calculations and SEM pictures
