# Supplementary material for: Guided immobilisation of single gold nanoparticles by chemical electron beam lithography
Source: Beilstein J Nanotechnol. 2013 May 31;4:336–44. doi: 10.3762/bjnano.4.39 (PMC3678398; doi:10.3762/bjnano.4.39)
Supplement: File 1 — Additional CAD-drawings, theoretical calculations and SEM pictures [file Beilstein_J_Nanotechnol-04-336-s001.pdf]

Supporting Information

for

Guided immobilisation of single gold nanoparticles by chemical electron beam lithography

Patrick A. Schaal and Ulrich Simon*

Address: Institute of Inorganic Chemistry and JARA – Fundamentals of Future Information Technology, RWTH Aachen University, Landoltweg 1, D-52056 Aachen, Germany

Email: Ulrich Simon* - ulrich.simon@ac.rwth-aachen.de

* Corresponding author

Additional CAD-drawings, theoretical calculations and SEM pictures

Dose-coloured designs with large circles used for lithographic SAM structuring

Both designs are composed of circles 400 nm or 200 nm in diameter. The spacing between the circles is 1 µm in the horizontal and vertical directions. In addition, the applied electron dose rises from bottom (0× base dose) to top (200× base dose) in steps of 5× base dose.

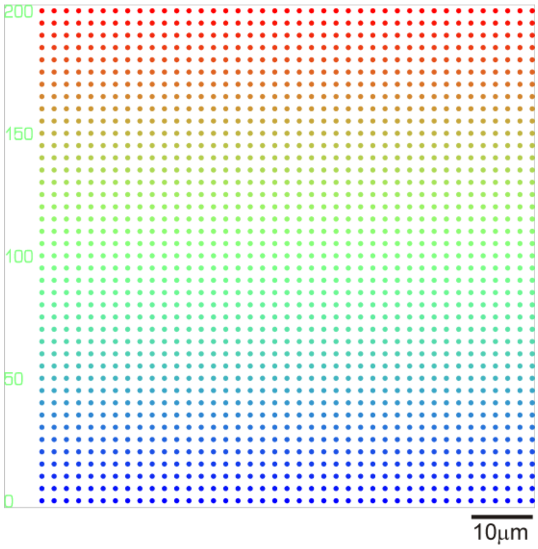

Figure S1: CAD-drawing of pattern with 400 nm circles.

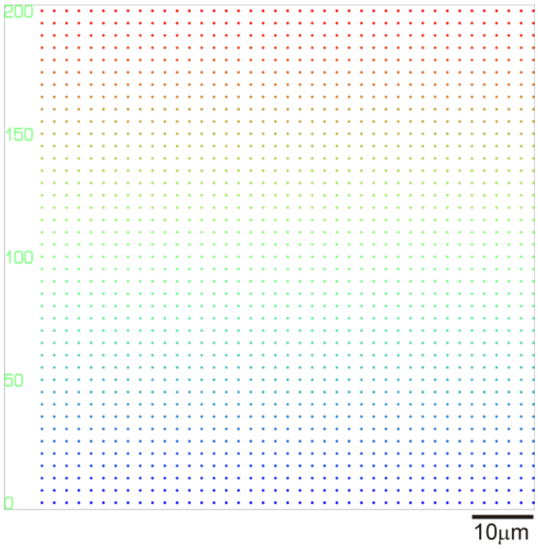

Figure S2: CAD-drawing of pattern with 200 nm circles.

Theoretical calculations of primary electron pathways

Theoretical calculations of the pathways of primary electrons (PE) at energies of 2 keV show that a fraction of electrons is able to pass through 40 nm of SiO_2 (see Figure S3). Nevertheless, it is not necessary to adjust the applied electron dose known from previous experiments, because the reduction is mainly induced by low energy secondary electrons (SEs) [1-5]. Since these low energy SEs can leave the substrate from a depth within the range of a just few nanometres, the thickness reduction of the SiO_2 layer does not need to be compensated [6].

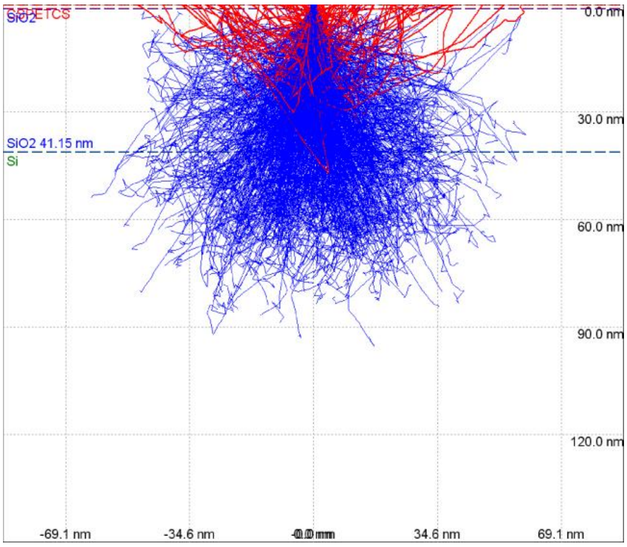

Figure S3: Monte Carlo simulation of primary electron paths within the used three-layered system (100 µm Si/40 nm SiO_2/1.3 nm CSPETCS SAM).

SEM images of SAM patterned with 100 nm circles after incubation with 16 nm AuNP

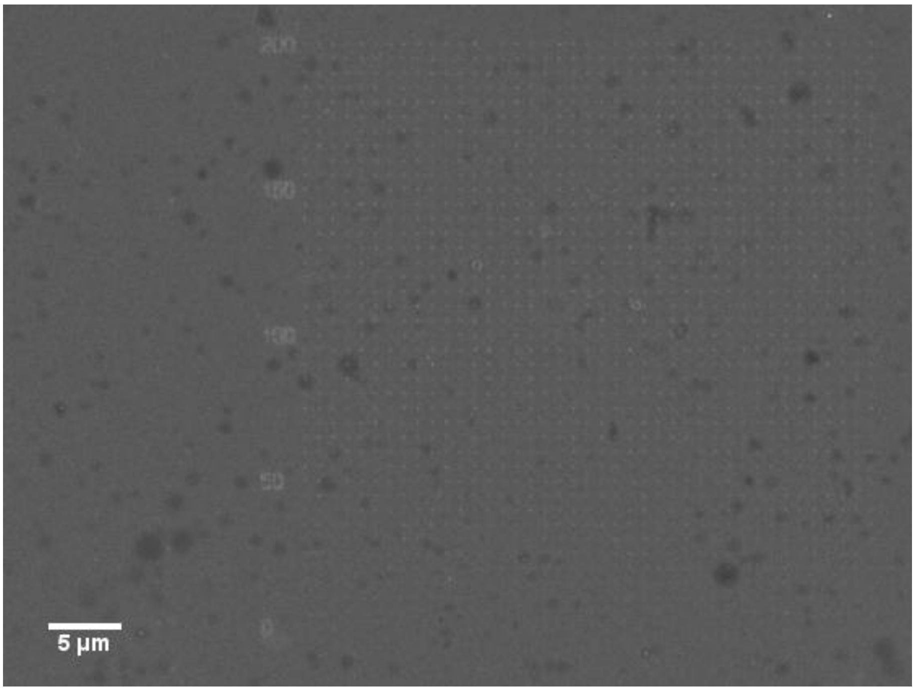

Figure S4: SEM image (overview) of a pattern with circles of 100 nm diameter, which was incubated with 16 nm AuNPs.

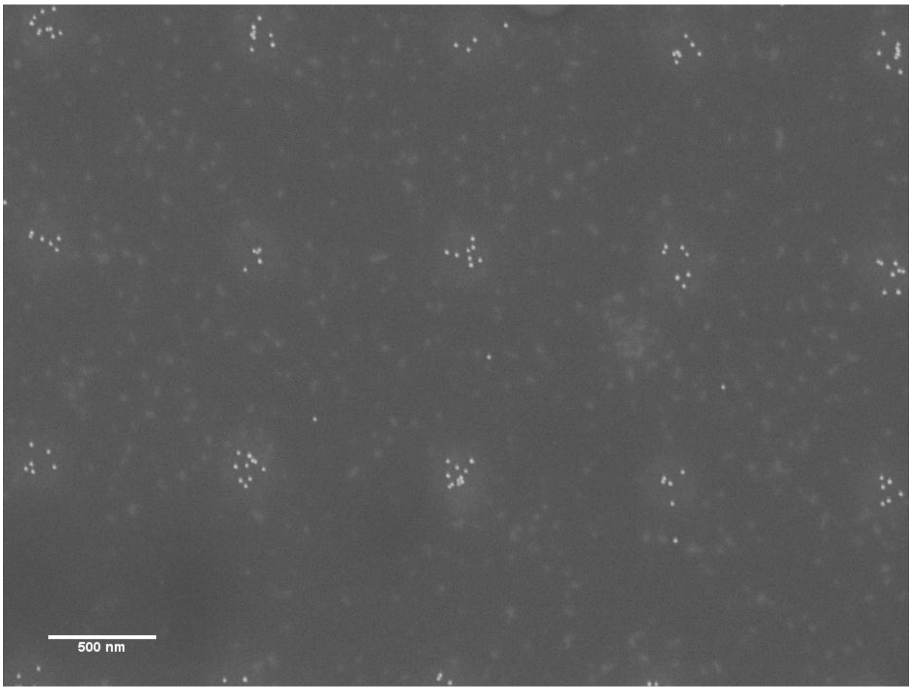

Figure S5: SEM image (zoom) of a pattern with circles of 100 nm diameter, which was incubated with 16 nm AuNPs.

Dose-coloured design for pointwise irradiation with single-exposure dots

This design uses 400 nm circles on the outside as a guide (pitch and dose scaling similar to the design shown in Figure S1). The middle part is composed of an array of single irradiation points (horizontal and vertical pitch 100 nm with dose scaling equal to outer guiding circles).

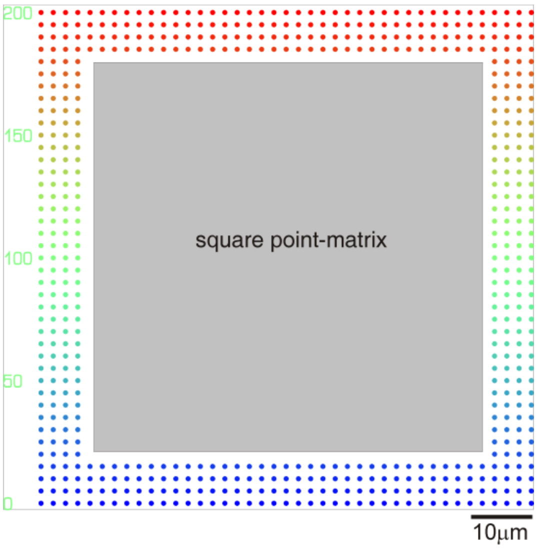

Figure S6: CAD-drawing of pattern with single-exposure dots.

Generation of thiol patterns on other oxides

In general, it is possible to silanise various other oxides, e.g., ITO, SnO_2, TiO_2, using wet-chemical approaches [7-12]. Therefore, we silanised ITO-coated flexible PET foils with CSPETCS, irradiated the samples with electrons (EHT = 2 kV and base dose of 10 $\mu C \cdot cm^{-2}$) and incubated them with a solution of citrate-stabilised 16 nm AuNP in order to generate AuNP patterns. Figure S7a and b shows SEM images of the sample after particle immobilisation. Although, the formed patterns are not as precise as the patterns shown for Si/SiO_2 substrates, one can see a preferred immobilisation of the AuNP into the intended pattern. This result is considered as a proof-of-principle for the general compatibility of this approach in generating AuNP patterns on oxides other than SiO_2, which can be functionalised with silanes as well.

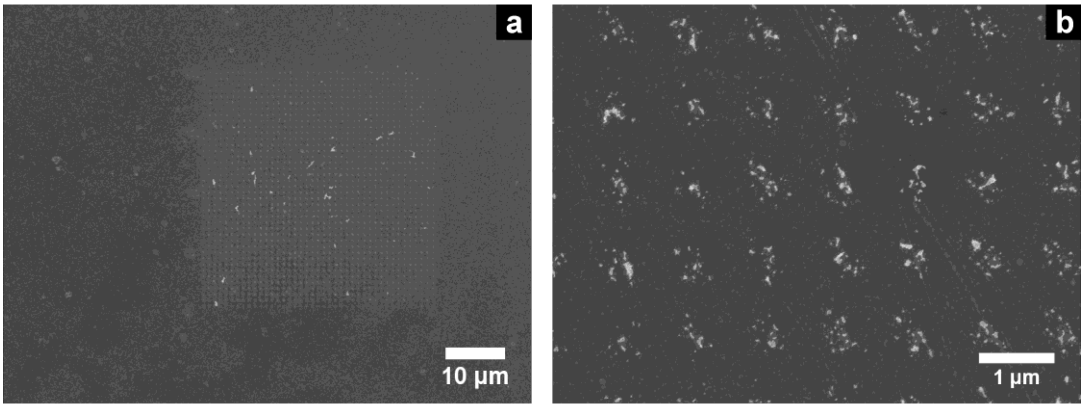

Figure S7: SEM images of silanised ITO-covered PET foils after electron irradiation and immobilisation of 16 nm AuNPs.

References

1. Chesneau, F.; Hamoudi, H.; Schüpbach, B.; Terfort, A.; Zharnikov, M. *J. Phys. Chem. C* **2011,** *115,* 4773–4782. doi:10.1021/jp111710x
2. Lud, S. Q.; Neppl, S.; Richter, G.; Bruno, P.; Gruen, D. M.; Jordan, R.; Feulner, P.; Stutzmann, M.; Garrido, J. A. *Langmuir* **2010,** *26,* 15895–15900. doi:10.1021/la102225r
3. Turchanin, A.; Käfer, D.; El-Desawy, M.; Wöll, C.; Witte, G.; Gölzhäuser, A. *Langmuir* **2009,** *25,* 7342–7352. doi:10.1021/la803538z
4. Iqbal, P.; Critchley, K.; Attwood, D.; Tunnicliffe, D.; Evans, S. D.; Preece, J. A. *Langmuir* **2008,** *24,* 13969–13976. doi:10.1021/la802244a
5. Turchanin, A.; Schnietz, M.; El-Desawy, M.; Solak, H. H.; David, C.; Gölzhäuser, A. *Small* **2007,** *3,* 2114–2119. doi:10.1002/smll.200700516
6. Reimer, L. In *Scanning Electron Microscopy - Physics of Image Formation and Microanalysis*, 2nd ed.; Hawkes, P. W., Ed.; Springer: 1998.
7. Neouze, M.-A.; Schubert, U. *Monatsh. Chem.* **2008,** *139,* 183–195. doi:10.1007/s00706-007-0775-2
8. Helmy, R.; Fadeev, A. Y. *Langmuir* **2002,** *18,* 8924–8928. doi:10.1021/la0262506
9. Markovich, I.; Mandler, D. *J. Electroanal. Chem.* **2001,** *500,* 453–460. doi:10.1016/S0022-0728(00)00458-7
10. Chen, K.; Caldwell, W. B.; Mirkin, C. A. *J. Am. Chem. Soc.* **1993,** *115,* 1193. doi:10.1021/ja00056a080
11. Untereker, D.; Lennox, J. C.; Wier, L.; Moses, P.; Murray, R. W. *J. Electroanal. Chem.* **1977,** *81,* 309–318. doi:10.1016/S0022-0728(77)80027-2
12. Moses, P. R.; Wier, L.; Murray, R. W. *Anal. Chem.* **1975,** *47,* 1882–1886. doi:10.1021/ac60362a043
